# Associations of *lymphotoxin-a* (*LTA*) rs909253 A/G gene polymorphism, plasma level and risk of ankylosing spondylitis in a Chinese Han population

**DOI:** 10.1038/s41598-020-57927-6

**Published:** 2020-01-29

**Authors:** Aiping Zhu, Zhicheng Yang, Hui Zhang, Ruiping Liu

**Affiliations:** 1 Department of Orthopaedics, The Affiliated Changzhou No.2 People’s Hospital of Nanjing Medical University, Changzhou, 213003 China; 2 Department of Orthopaedics, The Jintan Hospital Affiliated to Jiangsu University, Changzhou, 213200 China

**Keywords:** Genetics research, Ankylosing spondylitis

## Abstract

Lymphotoxin-a (LTA) may be associated with the pathogenesis of inflammatory diseases. To assess the association of the *LTA* rs909253 A/G polymorphism with plasma level and risk of ankylosing spondylitis (AS) in a Chinese Han population. Genotyping and LTA plasma were tested by mass spectroscopy and enzyme-linked immunosorbent assay (ELISA), respectively. The results showed that the average plasma level of LTA in AS was significantly lower than in the controls (*P* = 0.000). Our results also indicated that *LTA* rs909253 A/G was associated with a decreased risk of AS (G vs. A: *P* = 0.014). Significant differences were also found between the rs909253 A/G genotype and down-regulated plasma level in AS patients, compared with controls. After stratification analysis, a decreased risk of AS was associated with the *LTA* rs909253 G allele (G *vs*. A) among female patients, younger patients (Yr. < 30), HLA-B27-positive patients. In addition, In conclusion, *LTA* rs909253 A/G genotype has a significant relationship with decreased susceptibility to AS.

## Introduction

Ankylosing spondylitis (AS) is a common rheumatic chronic inflammatory disease. It often occurs in young men. It begins with the sacroiliac joint and then gradually causes stiffness and pain in the spine. In many patients, the bilateral hip joints are involved. AS often results in limited movement of the spine, sacroiliac joint and hip joint, which can cause serious limitations to the patients’ life and work^1^. Up to now, the pathogenesis of AS remains unclear. Immune response dysregulation, infectious agents, and genetic factors may cause the development of AS^2^. A twin study confirmed that AS susceptibility is largely determined by heredity, while HLA-B27 accounts for a small proportion of the whole genetic susceptibility^3^.

Lymphotoxin-a (LTA), another name is tumor necrosis factor-β (TNF-β), is a close homologue of tumor necrosis factor-α (TNF-a)^4^. The *TNF-α* gene is located on chromosome 6, and between *HLA-B* and *HLA-DR*^5^. LTA is a proinflammatory cytokine produced by lymphocytes that causes tissue injury. It also can significantly affect the function of lymphogenesis^6,7^. Lymphotoxin (LT) signaling plays a key role in lymphogenesis and maintenance^8^. Studies have shown that the inflammatory factors of IL-22 and IL-23 are associated with the development of AS^9,10^. Furthermore, IL-22 and IL-23 production for host defense were regulated by the LT pathway in adult innate lymphoid cells^11^. Therefore, LTA is correlated to the pathogenesis of inflammatory diseases.

The *LTA* rs909253 A/G polymorphism has been correlated to the risk of developing several autoimmune diseases, such as scleroderma^12^, multiple sclerosis^13^ and systemic lupus erythematosus^5,14^. Single nucleotide polymorphisms (SNPs) of the *LTA* gene have been suggested to be associated with the susceptibility of AS^15,16^. For example, a case-control study showed that rs909253 may influence susceptibility to AS^16^.

Recently, a study by Fabiano Aparecido de Medeiros *et al*. explored the association between the *LTA rs909253* polymorphism with plasma LTA level, the susceptibility for RA, and the presence of autoantibodies. They found that the *LTA rs909253* polymorphism was not correlated to RA susceptibility and LTA plasma levels. However, the B1 allele had significantly correlated to the presence of autoantibodies. Furthermore, interaction between the presence of autoantibodies and B1 allele has significantly related to the increase of plasma LTA level in RA patients^17^.

In this study, we investigated the potential correlation between the plasma level of LTA and AS, and examined associations between the plasma level of LTA and clinical parameters in the Chinese Han population. The correlations between rs909253 and plasma LTA level also have been tested. Finally, we tested the correlation between rs909253 and susceptibility to AS.

## Results

### Characteristics of the study population

The demographic and clinical characteristics of all subjects are summarized in Table 1. Subjects were adequately matched for age and sex (*P* = 0.345 and 0.815, respectively). The genotype distributions of *LTA* rs909253 A/G in all subjects are illustrated in Table 2. The observed genotype frequencies for the polymorphism in controls were in HWE for *LTA* rs909253 A/G (*P = *0.109).

**Table 1:** Patient demographics and risk factors in ankylosing spondylitis.

Variable*	Cases (n = 190)	Controls (n = 190)	*P*
Age (years)	32.49 (±10.15)	33.38 (±8.10)	0.345
Male/female	142/48	140/50	0.815
CRP positive, no. (%)	118 (62.11%)	NA	NA
HLA-B27 positive, no. (%)	151 (79.47%)	NA	NA
**Grading of sacroiliac joint, no. (%)**			
Grade I	0 (0.00%)	NA	NA
Grade II	136 (71.58%)	NA	NA
Grade III	31 (16.32%)	NA	NA
Grade IV	23 (12.11%)	NA	NA
AA + AG + GG LTA levels^∗∗^	61.20/109.80	5202.00/9333.00	**0.000**^∗∗∗^
AA (25/26) LTA levels	18.64/33.08	466.00/860.00	**0.001**^∗∗∗^
AG (44/37) LTA levels	28.80/55.51	1267.00/2054.00	**0.000**^∗∗∗^
GG (16/22) LTA levcls	15.38/22.50	5202.00/9333.00	0.052^∗∗∗^

*CRP: C-reactive protein. **LTA levels were available in 85 AS cases (AA: 26; AG: 37; GG: 22 of *LTA* rs909253 A/G) and 85 controls (AA: 25; AG: 44; GG: 16 of *LTA* rs909253 A/G), with age, *P* = 0.214; sex, *P* = 0.506 (cases vs. controls). ***P value was calculated by non-parametric tests. Bold values are statistically significant (*P* < 0.05).

**Table 2:** Logistic regression analysis of associations between *LTA* rs909253 A/G polymorphisms and risk of ankylosing spondylitis.

Genotype	Cases* (n = 190)		Controls (n = 190)		OR (95% CI)	*P*	OR (95% CI)	*P*
	n	%	n	%			Adjust^∗∗^	Adjust
AA	65	34.39	53	28.19	1.00	NA	1.00	NA
AG (AG vs. AA)	93	49.21	83	44.15	0.91 (0.57–1.46)	0.705	0.92 (0.57–1.47)	0.722
GG (GG vs. AA)	31	16.40	52	27.66	**0.49 (0.27–0.86)**	**0.014**	**0.46 (0.26–0.83)**	**0.010**
AG + GG vs. AA	NA	NA	NA	NA	0.75 (0.48–1.16)	0.195	0.74 (0.48–1.15)	0.185
GG vs. AG + AA	NA	NA	NA	NA	**0.51 (0.31–0.85)**	**0.009**	**0.50 (0.30–0.82)**	**0.007**
G vs. A	NA	NA	NA	NA	**0.70 (0.53–0.94)**	**0.016**	**0.70 (0.52–0.93)**	**0.014**

*The genotyping was successful in: 189 cases and 188 controls for *LTA* rs909253 A/G. **Adjusted by age and sex. Bold values are statistically significant (*P* < 0.05).

### Association between *LTA* rs909253 A/G Polymorphisms and the Risk of AS

Logistic regression analyses revealed that *LTA* rs909253 A/G polymorphism was associated with the risk of AS (Table 2). Using genotypes AA as a reference, genotype GG acted as a protection factor for AS patients (GG *vs*. AA: OR = 0.46, 95%CI = 0.26–0.83; *P* = 0.010). Using genotypes AG + AA as a reference, genotype GG acted as a protection factor for AS patients (GG *vs*. AG + AA: OR = 0.74, 95%CI = 0.48–1.15; *P* = 0.007). Our analysis also revealed that *LTA* rs909253 G allele was associated with significantly decreased risk of AS (G *vs*. A: OR = 0.70, 95%CI = 0.52–0.93; *P* = 0.014) than the rs909253 A allele in the Chinese Han population.

### Stratification analyses of *LTA* rs909253 A/G polymorphisms and the risk of RA

Stratification analyses were performed according to age, sex, HLA-B27 (Table 3). Following stratified analysis, a decreased risk of AS was associated with the *LTA* rs909253 G allele (G *vs*. A) among female patients (OR = 0.55, 95%CI = 0.31–0.97, *P* = 0.040), younger patients (Yr. < 30) (OR = 0.56, 95%CI = 0.35–0.89, *P* = 0.014), HLA-B27-positive patients (OR = 0.73, 95%CI = 0.54–0.99, *P* = 0.040).

**Table 3:** Stratified Analyses between *LTA* rs909253 A/G Polymorphisms and the Risk of Ankylosing Spondylitis.

Variable	*LTA rs909253 A/G* (case/control)				OR (95% CI); *P*			
	AA	AG	GG	G versus A	AG versus AA	GG versus AA	AG + GG versus AA	GG versus AG + AA
**Sex**								
Male	45/38	70/63	26/38	0.76 (0.55–1.06); 0.110	0.94 (0.54–1.63); 0.820	0.58 (0.30–1.12); 0.103	0.80 (0.48–1.34); 0.402	1.66 (0.95–2.93); 0.078
Female	20/15	23/20	5/14	**0.55 (0.31–0.97); 0.040**	0.86 (0.35–2.12); 0.747	**0.27 (0.08–0.91); 0.034**	0.62 (0.27–1.43); 0.258	**3.44 (1.13–10.48); 0.030**
Age (years)								
< 30	29/13	43/29	15/21	**0.56 (0.35–0.89); 0.014**	0.67 (0.30–1.49); 0.321	**0.32 (0.13–0.81); 0.017**	**0.52 (0.24–1.11); 0.090**	**2.40 (1.12–5.15); 0.025**
≥ 30	36/40	50/54	16/31	0.78 (0.53–1.13); 0.185	1.03 (0.57–1.86); 0.925	0.57 (0.27–1.22); 0.148	0.86 (0.50–1.50); 0.601	1.77 (0.91–3.47); 0.094
HLA-B27								
Negative	13/53	19/83	4/52	0.61 (0.36–1.02); 0.059	0.93 (0.43–2.05); 0.863	0.31 (0.10–1.03); 0.055	0.70 (0.33–1.47); 0.341	**3.06 (1.03–0.08); 0.044**
Positive	52/53	74/83	27/52	**0.73 (0.54–0.99); 0.040**	0.91 (0.55–1.49); 0.704	0.53 (0.29–0.97); 0.038	0.76 (0.48–1.21); 0.249	**1.78 (1.06–3.01); 0.030**

Bold values are statistically significant (*P* < 0.05).

### Production of LTA in AS Patients, Controls and Different Genotypes

**Figure 1:**
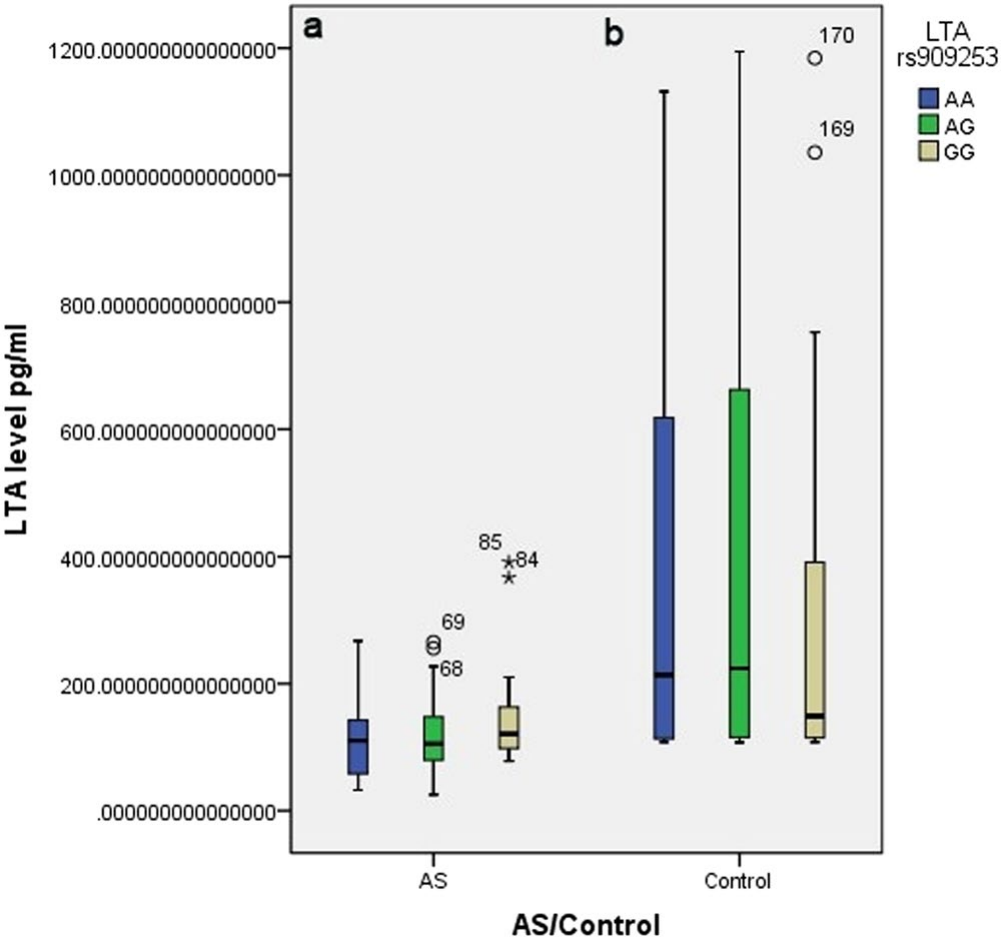
Association between LTA levels and LTA rs909253 A/G genotype frequencies in (**a**) ankylosing spondylitis patients and (**b**) controls.

The average plasma concentration of LTA was significantly lower in AS patients compared with controls (Table 1). We compared LTA plasma levels on basis of *LTA* rs909253 A/G genotypes. We found *LTA* rs909253 A/G genotypes had significant lower levels of LTA in AS patients when compared with control groups except for group genotype GG (Table 1). However, we did not find the significant statistic associations between LTA plasma levels and different *LTA* rs909253 A/G genotypes in AS patients or control groups (Fig. 1).

### Stratification of association between plasma level of LTA and other biomarkers

Our study indicated the LTA plasma levels of female AS patients were significantly lower than male AS patients. However, no associations were obtained between plasma levels of LTA and sex, age, HLA-B27, C-reactive protein (CRP), or grade of the sacroiliac joint in AS patients (Table 4).

**Table 4:** Stratification of association between plasma levels of LTA and other biomarkers in ankylosing spondylitis patients.

Variable		Case, n	P*
Age	≥30Ys	54	0.493
	<30Ys	31	
Sex	Male	61	0.109
	Female	24	
HLA-B27	Negative	25	0.539
	Positive	60	
CRP status	Negative	35	0.237
	Positive	50	
Grading of Sacroiliac joint	I + II	58	0.902
	III + IV	27	

*P value was calculated by non-parametric tests. Bold values are statistically significant (*P* < 0.05).

### Combined analysis with recent researches of LTA rs909253 A/G polymorphisms in AS

Combined with recent two other researches found that *LTA* rs909253 AG increased risk of AS significantly than controls (OR = 1.28; 95% CI = 1.01–1.62; *P* = 0.038) (Table 5).

**Table 5:** Meta-analysis of the association between *LTA* rs909253 A/G polymorphisms and ankylosing spondylitis risk.

SNP	Comparison	Category	Category	OR (95% CI)	*P*-value	*P* for heterogeneity
*LTA* rs909253	G vs. A	Total		1.20(0.70, 2.07)	0.512	0.000
		2019	This study	0.70(0.53, 0.94)	0.016	NA
		2017	Jia B	1.53(1.07, 2.18)	0.020	NA
		2011	Chen J	1.62(1.26, 2.08)	0.000	NA
	GG vs. AG + AA	Total		1.38(0.46, 4.12)	0.564	0.000
		2019	This study	0.51(0.31, 0.85)	0.009	NA
		2017	Jia B	2.15(1.02, 4.52	0.043	NA
		2011	Chen J	2.54(1.33, 8.45)	0.005	NA
	GG + AG vs. AA	Total		1.25(0.77, 2.04)	0.373	0.012
		2019	This study	0.75(0.48, 1.16)	0.195	NA
		2017	Jia B	1.53(0.95, 2.45)	0.080	NA
		2011	Chen J	1.65(1.21, 2.24)	0.001	NA
	GG vs. AA	Total		1.50(0.45, 5.02)	0.513	0.000
		2019	This study	0.49(0.27, 0.86)	0.014	NA
		2017	Jia B	2.46(1.13, 5.35)	0.024	NA
		2011	Chen J	2.95(1.53, 5.70)	0.001	NA
	AG vs. AA	Total		1.28(1.01, 1.62)	0.120	0.241
		2019	This study	0.91(0.57, 1.46)	0.705	NA
		2017	Jia B	1.33(0.80, 2.20)	0.273	NA
		2011	Chen J	1.49(1.08, 2.06)	0.016	NA

Bold values are statistically significant of total values (*P* < 0.05).

## Discussion

In the current case-control association study, our present data suggest that the *LTA* rs909253 A/G genotype is associated with decreased susceptibility to AS. In addition, we also found *LTA* rs909253 A/G genotypes had significant lower levels of LTA in AS patients compared to control groups. In stratification analysis, we found a decreased risk of AS was associated with the *LTA* rs909253 G allele (G *vs*. A) among female patients, younger patients (Yr. < 30) and HLA-B27-positive patients.

AS can lead to a decrease in the quality of life of patients. However, there is no cure for AS, although treatments and medications can reduce symptoms and pain. Many researchers want to find new ways to prevent and treat AS. Genetic factors may contribute to the development of AS^2^. Approximately 90% of people with AS are the *HLA-B27* genotype, and thus, there is a strong genetic association^18^. However, only 1–2% of the persons with the *HLA-B27* genotype develop AS. Investigating AS-related genetic factors may be helpful in the prevention and diagnosis of AS. Thus, we explored the associations between the *LTA* rs909253 A/G polymorphism, plasma level and risk of AS in the Chinese Han population.

*LTA* is located at the HLA-III region of chromosome 6p, is closely linked to *TNF-a*. *LTA* gene consists of four exons and three introns. LTA plays a critical role in inflammatory regulation, anti-virus response and immune activation, similar to TNF-α^19,20^.

There have been studies on rs909253 and gastric cancer, chronic obstructive pulmonary disease (COPD), and coronary heart diseases^21–23^. A meta-analysis suggested that rs909253 was correlated with the risk of gastric cancer, and especially in Asians^21^. However, no significantly different genotype frequencies of rs909253 were seen in COPD or coronary heart disease compared with controls^22,23^. In this study, we found that rs909253 was correlated with a decreased risk of AS (G vs. A: OR = 0.70, 95%CI = 0.52–0.93; *P* = 0.014). Then, we searched PubMed and meta-analyzed our results with the results of two other studies on this locus^15,16^. We found that rs909253 had no risk of AS.

LTA is a soluble protein released by lymphocytes and activated by antigens or mitogens. It can inhibit the activity of tumor cells^24^. Bachmann believed that blocking lymphotoxin might be a promising therapeutic strategy for other autoimmune diseases, such as Hashimoto’s thyroiditis and arthritis^25^. As far as we know, there have not been any studies on the level of LTA in AS. Thus, we tested the plasma LTA level in AS and healthy controls. We found that the average plasma level of LTA was significantly lower in AS patients, compared with controls (*P = *0.000).

We stratified the plasma LTA levels of the AS and control groups according to rs909253 genotype. We found that the LTA levels of the genotypes were significantly lower in the AS group except for group genotype GG (Table 1). Recently, Bolstad AI *et al*. investigated whether SNPs in the *LTA* gene clusters were correlated with primary Sjogren ‘s syndrome, and they found that *LTA* rs909253 and rs1800629 had significantly association with primary Sjogren’s syndrome, and the correlations were mainly due to anti-Ro/SSA and anti-La/SSB antibody-positive primary Sjogren’s syndrome^26^. Therefore, we hypothesized that *LTA* rs909253 may affect mechanistic pathways in AS. We will do functional studies of LTA rs909253 in our future research.

Our study has some limitations. First, because of our hospital-based case–control design, we may have selection bias. Second, we investigated SNPs just based on the functional characteristics, another fine-mapping study is required. Third, we use a medium sample size, so our analytical power is limited, although we also combined the results with other two independent studies. Forth, the control group we recruited was trauma patients, which may have a bias on our study. We will collect healthy patients as control in the future research. Fifth, because of the bias of choice, there is a slight difference in the proportion of HLA-B27 positive patients between the 85 vs. 85 samples and the 190 vs. 190 samples. However, because we use a medium sample size, studies including larger population, more ethnic groups are required.

## Materials and Methods

### Subjects

We obtained approval of the study protocol from the Ethics Committee of Nanjing Medical University (Nanjing, China). All patients provided written informed consent to be included in the study. We confirmed that all research was performed in accordance with relevant guidelines. One hundred and ninety AS patients were consecutively recruited from the Affiliated Changzhou No.2 People’s Hospital of Nanjing Medical University (Changzhou, China), the Changzhou First Hospital (Changzhou, China), between September 2010 and January 2016. A diagnosis of AS was established by using the classification criteria reported by the American College of Rheumatology (Modified New York Criteria)^27^. One hundred and ninety controls were traumatic patients without AS, matched AS for age (±5 years) and sex, and recruited from the same institutions during the same period time. Each patient was interviewed by trained personnel using a pre-tested questionnaire to obtain information on demographic data and related risk factors for AS. After the interview, 2 ml of peripheral blood was collected from each subject. Blood samples were collected using vacutainers and transferred to test tubes containing ethylenediamine tetra-acetic acid (EDTA).

Genomic DNA was isolated from whole blood using the QIAamp DNA Blood Mini Kit (Qiagen, Hilden, Germany). Genotyping was done by matrix-assisted laser desorption/ionization time-of-flight mass spectrometry (MALDI-TOF MS) using the MassARRAY system as previously described^28^.

The blood plasma concentration of LTA in 85 AS patients and 85 randomly selected controls using an enzyme-linked immunosorbent assay Kit (Boster, Wuhan, China). All analytical steps were performed in accordance with the manufacturer’s recommendations. The concentration of LTA was calculated by referring to a standard curve, according to the manufacturer’s instructions.

### Statistical analyses

Differences in demographics, variables, and genotypes of *LTA* rs909253 A/G polymorphism variants were evaluated using a chi-squared test. The associations between *LTA* rs909253 A/G genotypes and risk of AS were estimated by computing odds ratios (ORs) and 95% confidence intervals (CIs) using logistic regression analyses, and by using crude ORs. The Hardy–Weinberg equilibrium (HWE) was tested by a goodness-of-fit chi-squared test to compare the observed genotype frequencies to the expected frequencies among controls. Differences in *LTA* gene polymorphism and LTA blood plasma concentrations were evaluated using the Non parametric Tests. All statistical analyses were done with SAS software (version 9.1.3; SAS Institute, Cary, NC, USA).
